# Evaluating the Antiplasmodial Activity of *Picrasma javanica* Stem Bark Extract and Its Synergy With Artesunate in Rodent Malaria Models

**DOI:** 10.1155/sci5/7344444

**Published:** 2025-06-04

**Authors:** Sakaewan Ounjaijean, Voravuth Somsak

**Affiliations:** ^1^Research Institute for Health Sciences, Chiang Mai University, Chiang Mai 50200, Thailand; ^2^School of Allied Health Sciences, Walailak University, Nakhon Si Thammarat 80160, Thailand; ^3^Research Excellence Center for Innovation and Health Products, Walailak University, Nakhon Si Thammarat 80160, Thailand

**Keywords:** antiplasmodial activity, artesunate, *Picrasma javanica*, rodent malaria

## Abstract

Malaria remains a global health challenge, exacerbated by the emergence of drug-resistant *Plasmodium* strains. This study evaluates the antimalarial activity of *Picrasma javanica* stem bark extract and its combination with artesunate (ART) in *Plasmodium berghei*–infected mice. The extraction yield using hexane at a 1:5 (w/v) ratio was 7.8% w/w. Acute toxicity assessment indicated no mortality or adverse effects at doses up to 2000 mg/kg. In suppressive activity tests, *P. javanica* stem bark extract exhibited dose-dependent efficacy, achieving 13.3%, 36.4%, and 52.5% parasitemia inhibition at doses of 100, 200, and 400 mg/kg, respectively, with significant inhibition at higher doses (*p* < 0.05 and *p* < 0.01). ART showed 90.7% suppression (*p* < 0.001). In curative tests, the extract at 400 mg/kg reduced parasitemia by 31.0% (*p* < 0.05), while ART achieved 75.3% suppression (*p* < 0.001). The effective dose (ED_50_) values were 404.9 mg/kg for *P. javanica* stem bark extract and 2.03 mg/kg for ART. Combination treatments at ED_50_ ratios of 40/60 and 20/80 (extract/ART) resulted in 65.9% (*p* < 0.01) and 81.7% (*p* < 0.001) inhibition, respectively, with the 20/80 ratio showing a significant synergistic interaction (CI = 0.44121). The mean survival time (MST) for the untreated group was 12.2 ± 1.9 days. *P. javanica* stem bark extract and ART alone extended MST to 21.4 ± 2.4 and 21.8 ± 2.4 days, respectively (*p* < 0.05), while the 20/80 combination achieved the longest MST of 28.6 ± 1.7 days (*p* < 0.01; *p* < 0.05 vs. monotherapies). These findings highlight the potential of *P. javanica* stem bark extract as a complementary antimalarial agent when used with ART, offering a promising approach for combating drug-resistant malaria.

## 1. Introduction

Malaria remains a significant global health challenge, particularly in tropical and subtropical regions. According to the World Health Organization's (WHO) World Malaria Report 2023, there were an estimated 249 million malaria cases worldwide in 2022, marking an increase of five million cases compared to those in 2021. The disease also accounted for approximately 597,000 deaths in 2022, with the African region bearing the highest burden, comprising 94% of cases and 95% of deaths [1]. These statistics underscore the persistent threat malaria poses to global health, despite ongoing control and elimination efforts. Malaria is caused by protozoan parasites of the genus *Plasmodium*, transmitted to humans through the bites of infected female *Anopheles* mosquitoes. Among the five *Plasmodium* species known to infect humans, *P. falciparum* is responsible for the most severe and fatal cases, while *P. vivax* is associated with recurrent infections [2]. The rodent malaria parasite *Plasmodium berghei* serves as a valuable model organism for studying malaria biology and evaluating potential antimalarial interventions due to its genetic similarity to human-infecting species and its amenability to laboratory manipulation [3]. The emergence and spread of antimalarial drug resistance present critical challenges to malaria control efforts. Resistance to conventional drugs such as chloroquine and sulfadoxine-pyrimethamine has rendered these treatments ineffective in many endemic regions. More recently, partial resistance to artemisinin, the cornerstone of artemisinin-based combination therapy (ACT), has been documented in Southeast Asia and Africa [4]. ACTs, which pair artemisinin derivatives with partner drugs, remain the standard treatment for uncomplicated malaria; however, resistance to both components could severely undermine their efficacy [5, 6]. This situation necessitates the urgent development of new antimalarial agents with novel mechanisms of action.

Medicinal plants have historically been a rich source of antimalarial compounds, exemplified by the discovery of quinine from *Cinchona* bark and artemisinin from *Artemisia annua*. Research into plant-derived extracts and phytochemicals continues to yield promising leads for antimalarial drug development [7]. These natural products often possess diverse chemical structures and mechanisms, making them valuable in overcoming resistance. Several plant species have demonstrated potent antimalarial activity, and their traditional use in treating febrile illnesses highlights their therapeutic potential [8]. *Picrasma javanica*, a species in the Simaroubaceae family, is traditionally used in Southeast Asia for its medicinal properties [9]. Phytochemical investigations have identified various bioactive compounds in *P. javanica*, including quassinoids, alkaloids, and flavonoids, which exhibit a wide range of pharmacological activities such as anti-inflammatory, antipyretic, antimicrobial, and cytotoxic effects [10, 11]. These findings suggest its potential as a source of therapeutic agents. Previous studies have reported the antimalarial activity of *P. javanica* against *Plasmodium* species. Stem bark extracts of *P. javanica* have demonstrated significant in vitro antimalarial activity, likely attributed to the presence of quassinoids, a class of compounds known for their potent antimalarial properties [12]. However, comprehensive studies on its efficacy in vivo model and in combination with standard antimalarial drugs, such as artesunate (ART), remain limited. Understanding the potential synergistic effects of such combinations could provide new insights into overcoming drug resistance and enhancing therapeutic efficacy.

Hence, this study aims to evaluate the antimalarial activity of *P. javanica* stem bark extract in *P. berghei–*infected mice and to investigate its potential in combination therapy with ART. The findings may contribute to the development of alternative or adjunctive strategies for combating malaria in the context of increasing drug resistance.

## 2. Materials and Methods

### 2.1. Plant Material

The stem bark of *Picrasma javanica* was collected from the Garden of Medicinal Plants at the Faculty of Pharmacy, Chiang Mai University, Chiang Mai, Thailand, in May 2024. The plant material was authenticated by a botanist based on morphological and taxonomic characteristics. A voucher specimen (Voucher No. PJ-WU-752567) was prepared and deposited at the Herbarium of the Research Institute, Western University, Thailand, for future reference. The stem bark was thoroughly cleaned to remove debris, air-dried at room temperature, and finely powdered using a mechanical grinder. The powdered material was then stored in an airtight container at room temperature until further processing. This preparation ensured the preservation of phytochemical integrity for subsequent extraction and evaluation.

### 2.2. Preparation of Crude Extract

The crude extract of *Picrasma javanica* stem bark was prepared using a maceration method with hexane as the solvent [12]. Air-dried and powdered stem bark (approximately 500 g) was soaked in hexane at a 1:5 (w/v) ratio for 72 h at room temperature with occasional stirring. The mixture was then filtered through Whatman No. 1 filter paper, and the plant residue was re-extracted twice under identical conditions to maximize the recovery of bioactive compounds. The combined hexane filtrates were concentrated under reduced pressure using a rotary evaporator at 40°C to remove the solvent. The resulting crude extract was further dried in a vacuum desiccator to obtain a consistent dry powder. The total weight of the dried extract was recorded to calculate the extraction yield. The *P. javanica* stem bark extract was then stored in an airtight container at 4°C until further use. Prior to administration, the extract was dissolved in distilled water containing 0.5% carboxymethylcellulose (CMC) to form a uniform suspension. The suspension was freshly prepared daily at the required concentration for oral gavage to ensure stability and reproducibility of dosing. This preparation preserved the extract's bioactivity for subsequent antimalarial evaluations.

### 2.3. Preparation of Standard Antimalarial Drug

ART, with a purity greater than 98%, obtained from Sigma-Aldrich (Product Number: A3731), was used as the standard antimalarial drug in this study. The drug was freshly prepared each day to ensure stability and accuracy during administration. ART was dissolved in distilled water containing 0.5% CMC to produce a homogeneous suspension suitable for oral administration. The final concentration of the ART suspension was adjusted to deliver the intended dosage based on the body weight of the experimental mice (mg/kg). The administration volume was standardized at 10 mL/kg body weight to minimize variability and ensure accurate dosing. The suspension was thoroughly mixed before each use to prevent sedimentation and to ensure uniform drug distribution.

For combination therapy, ART was co-administered with *P. javanica* stem bark extract. Both agents were prepared separately and administered sequentially via oral gavage to ensure precise dosing and to maintain consistency throughout the experimental procedures.

### 2.4. Experimental Mice

Male Balb/c mice, aged 6–8 weeks and weighing approximately 20–25 g, were used in this study. The animals were obtained from a certified supplier (Nomura Siam International Co., Ltd.) and were acclimatized for 1 week prior to the start of the experiments. They were housed under standard laboratory conditions, including a controlled environment with a 12-h light/dark cycle, ambient temperature of 22°C–24°C, and relative humidity of 50%–60%. The mice had *ad libitum* access to standard rodent chow and water throughout the study period. All experimental procedures were conducted in accordance with the ethical guidelines outlined by the National Research Council of Thailand and were approved by the Animal Ethics Committee of Western University, Thailand (Approval Number: WTU2024-AE23). Efforts were made to minimize animal distress and discomfort, in line with international standards for the humane care and use of laboratory animals.

The selection of male Balb/c mice was based on their well-established susceptibility to *Plasmodium berghei* infection, ensuring the reproducibility and reliability of antimalarial activity assessments. The health and behavior of the animals were monitored daily, and any signs of illness or distress were promptly addressed by veterinary personnel.

### 2.5. Rodent Malaria Parasite

The rodent malaria parasite *Plasmodium berghei* ANKA (PbANKA) was used for infection in this study. The parasite strain was obtained from the Malaria Research and Reference Reagent Resource Center (MR4) and maintained as cryopreserved stocks in liquid nitrogen. Prior to experimental use, the parasites were thawed and passaged mechanically through donor mice to ensure viability and infectivity. For experimental infections, donor mice with parasitemia levels exceeding 20% were anesthetized with isoflurane before blood collection. Isoflurane anesthesia was administered using an induction chamber with a flow rate of 2%–3% isoflurane in oxygen until the mice were fully sedated. Anesthesia was maintained during blood collection using a nose cone delivering 1%–2% isoflurane. The depth of anesthesia was monitored by assessing the absence of reflex responses, such as pedal withdrawal reflex. This approach ensured humane handling of the animals and minimized stress. Blood was collected via cardiac puncture using heparinized syringes. The collected blood was diluted with sterile phosphate-buffered saline (PBS) to achieve the desired inoculum. Each experimental mouse was inoculated intraperitoneally (IP) with 0.2 mL of the diluted blood suspension containing approximately 1 × 10^7^ infected red blood cells (iRBCs) [13].

Parasitemia was monitored daily by preparing thin blood smears from the tail vein. The smears were stained with Giemsa and examined under a light microscope using an oil immersion objective. The percentage parasitemia was calculated using the following formula:

(1) $$\begin{array}{c} \text{Parasitemia}\left(\%\right)=\frac{\left(\text{Number of iRBCs}\right)\times 100}{\text{Total of red blood cells counted}}. \end{array}$$

A minimum of 1000 red blood cells were counted per smear to ensure accuracy. This approach enabled precise evaluation of parasite progression and the efficacy of the antimalarial treatments under investigation in parasitemia estimation.

### 2.6. Assessment of Acute Toxicity

The acute toxicity of *P. javanica* stem bark extract was evaluated in accordance with the Organization for Economic Cooperation and Development (OECD) guideline 423 (Acute Oral Toxicity–Acute Toxic Class Method) [14]. Healthy male Balb/c mice, aged 6–8 weeks and weighing 20–25 g, were randomly selected for the study. The mice were fasted for 4 h prior to dosing, with free access to water, and were administered a single oral dose of the extract suspended in distilled water containing 0.5% CMC. An initial dose of 300 mg/kg body weight was administered to three mice. Observations for signs of toxicity, such as changes in behavior, physical appearance, motor activity, and mortality, were made at 30 min, 4 h, 24 h, and daily for 14 days. If no mortality was observed, the dose was increased to 2000 mg/kg and tested in an additional group of three mice under the same conditions. This protocol ensured a comprehensive evaluation of the acute toxicity profile of the extract, and provided a safety reference for its therapeutic application.

### 2.7. Suppressive Antimalarial Activity

The suppressive antimalarial activity of *P. javanica* stem bark extract was evaluated using the 4-day suppressive test as described by Peters et al. [15]. Male Balb/c mice, aged 6–8 weeks and weighing 20–25 g, were inoculated IP with 1 × 10^7^ iRBCs of PbANKA on Day 0. The animals were randomly assigned into five groups (*n* = 5 per group): a negative control group receiving vehicle (0.5% CMC in distilled water), three treatment groups receiving *P. javanica* stem bark extract at doses of 100, 200, and 400 mg/kg, respectively, and a positive control group treated with ART at a single dose of 10 mg/kg. All treatments were administered orally once daily for four consecutive days (Days 0–3) at a dosing volume of 10 mL/kg body weight. On Day 4, parasitemia was assessed using thin blood smears stained with Giemsa and examined under a light microscope. Parasitemia was calculated as the percentage of iRBCs among at least 1000 counted cells, and the percent inhibition of parasitemia was calculated relative to the negative control, using the following formula:

(2) $$\begin{array}{c} \text{Inhibition}\left(\%\right)=\frac{\left(\text{Parasitemia in negative control}-\text{Parasitemia in treated group}\right)}{\text{Parasitemia in negative control}}\times 100. \end{array}$$

### 2.8. Curative Antimalarial Activity

The curative antimalarial activity of *P. javanica* stem bark extract was assessed using the 4-day established infection model [16]. Male Balb/c mice, aged 6–8 weeks and weighing 20–25 g, were inoculated IP with 1 × 10^7^ iRBCs of PbANKA on Day 0. On Day 3 post-infection, when parasitemia was well established, the mice were randomly assigned to five groups (*n* = 5 per group): a negative control group receiving vehicle (0.5% CMC in distilled water), three treatment groups receiving *P. javanica* stem bark extract at doses of 100, 200, and 400 mg/kg, respectively, and a positive control group treated with ART at a dose of 10 mg/kg. All treatments were administered orally once daily for five consecutive days (Days 3–7) using a dosing volume of 10 mL/kg body weight. Thin blood smears were prepared from the tail vein of each mouse on Days 3, 5, and 7 post-infection to monitor parasitemia progression. The smears were stained with Giemsa and examined under a light microscope. Parasitemia was calculated as the percentage of iRBCs among at least 1000 counted cells. The efficacy of the treatments was expressed as the percentage reduction in parasitemia relative to the negative control group, using the following formula:

(3) $$\begin{array}{c} \text{Reduction}\left(\%\right)=\frac{\left(\text{Parasitemia in negative control}-\text{Parasitemia in treated group}\right)}{\text{Parasitemia in negative control}}\times 100. \end{array}$$

### 2.9. Assessment of Effective Doses

The effective doses of *P. javanica* stem bark extract and ART were evaluated using the 4-day suppressive test, as previously described [15]. Male Balb/c mice, aged 6–8 weeks and weighing 20–25 g, were inoculated IP with 1 × 10^7^ iRBCs of PbANKA on Day 0. The mice were randomly assigned to groups (*n* = 5 per group) and treated orally once daily for four consecutive days (Days 0–3) with varying doses of *P. javanica* stem bark extract (25, 50, 100, 200, 400, and 800 mg/kg) or ART (0.1, 1, 5, 10, and 20 mg/kg). A negative control group received the vehicle (0.5% CMC in distilled water). The dosing volume for all treatments was standardized at 10 mL/kg body weight. On Day 4, parasitemia was assessed by preparing thin blood smears from tail vein samples, staining them with Giemsa, and examining them under a light microscope. Parasitemia (%) was calculated as the percentage of iRBCs among at least 1000 counted cells. The suppressive effect of each dose was calculated as the percentage inhibition of parasitemia relative to the negative control group. Dose–response curves were generated for *P. javanica* stem bark extract and ART, and the effective dose required to suppress 50% of parasitemia (ED_50_) was determined using nonlinear regression analysis.

### 2.10. Antimalarial Activity of Combination Treatment

The antimalarial activity of the combination of *P. javanica* stem bark extract and ART was evaluated using the in vivo fixed ratios technique, as described by Nateghpour et al. [17]. Male Balb/c mice, aged 6–8 weeks and weighing 20–25 g, were inoculated IP with 1 × 10^7^ iRBCs of PbANKA on Day 0. The effective doses (ED_50_) of *P. javanica* stem bark extract and ART were determined from prior dose–response experiments. Based on the ED_50_ values, the combination treatments were prepared in fixed ratios of 100/0, 80/20, 60/40, 40/60, 20/80, and 0/100 (*P. javanica* stem bark extract/ART). Each treatment was calculated to deliver doses proportional to the ED_50_ values of both substances. The mice were randomly assigned into six groups (*n* = 5 per group) corresponding to the six fixed ratios. Treatments were administered orally once daily for four consecutive days (Days 0–3) at a dosing volume of 10 mL/kg body weight. A negative control group received the vehicles (0.5% CMC in distilled water). Parasitemia was assessed on Day 4 by preparing thin blood smears from tail vein samples, staining them with Giemsa, and examining them under a light microscope. Parasitemia was calculated as the percentage of iRBCs among at least 1000 counted cells. The percentage inhibition of parasitemia for each combination was calculated.

The interaction between *P. javanica* stem bark extract and ART was analyzed using CompuSyn software. Combination index (CI) values were calculated for each combination to classify the interaction as synergistic (CI < 1), additive (CI = 1), or antagonistic (CI > 1). This analysis provided insights into the combined antimalarial efficacy of *P. javanica* stem bark extract and ART.

### 2.11. Assessment of Mean Survival Time

The mean survival time (MST) of PbANKA-infected mice was assessed over a 30-day observation period to assess the effectiveness of treatments in prolonging survival. The day of death for each mouse was recorded, and the MST for each group was calculated using the following formula:

(4) $$\begin{array}{c} \text{MST}\left(\text{days}\right)=\frac{\text{Sum of survival days for all mice in the group}}{\text{Total number of mice in the group}}. \end{array}$$

### 2.12. Statistical Analysis

All data were analyzed using GraphPad Prism (version 10.2.2; GraphPad Software, USA) and CompuSyn software (ComboSyn, Inc., USA) for CI analysis. Results were expressed as the mean ± standard error of the mean (SEM). Comparisons between groups were performed using one-way analysis of variance (ANOVA) followed by Tukey's post hoc test to determine significant differences among treatment groups. A *p* value of < 0.05 was considered statistically significant. Dose–response relationships for *P. javanica* stem bark extract, ART, and their combinations were analyzed using nonlinear regression analysis. The ED_50_ values (best-fit) were determined using the sigmoidal dose–response curves with a variable slop model.

## 3. Results

### 3.1. Extraction Yield of *P. javanica* Stem Bark Extract

The stem bark of *P. javanica* was subjected to hexane extraction at a ratio of 1:5 (w/v). Following solvent evaporation, a dry crude extract was obtained, yielding an average of 7.8% (w/w). This yield was calculated based on the initial dry weight of the stem bark (500 g) and the final weight of the dried extract (39 g). The extraction procedure was conducted under controlled conditions to ensure the reproducibility and efficiency of compound recovery.

### 3.2. Acute Toxicity Assessment of *P. javanica* Stem Bark Extract in Mice

Oral administration of *P. javanica* stem bark extract at doses of 300 and 2000 mg/kg in mice resulted in no observable signs of toxicity or behavioral abnormalities during the 14-day observation period. Key parameters, including food and water intake, locomotor activity, grooming behavior, and general physical appearance, remained within normal limits. Furthermore, no mortality was recorded in any of the treatment groups throughout the study. These findings suggest that the *P. javanica* stem bark extract is well tolerated and nontoxic at doses up to 2000 mg/kg when administered orally, indicating a wide safety margin for further pharmacological investigations.

### 3.3. Suppressive Antimalarial Activity of *P. javanica* Stem Bark Extract Against PbANKA

**Figure 1:**
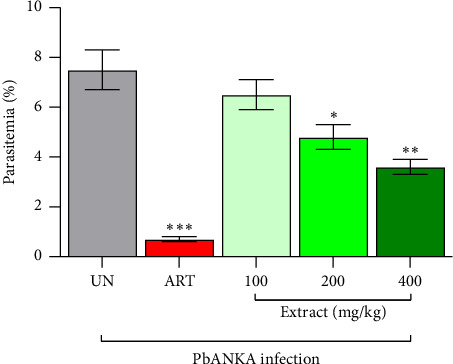
Suppressive antimalarial activity of *P. javanica* stem bark extract against PbANKA-infected mice. Mice were inoculated intraperitoneally with 1 × 10^7^ iRBCs of PbANKA on day 0 to establish infection. Oral treatments were administered once daily for four consecutive days (days 0–3). Treatment groups included *P. javanica* stem bark extract at doses of 100, 200, and 400 mg/kg, and ART at a dose of 10 mg/kg. The untreated group (UN) received the vehicle (0.5% CMC). Parasitemia levels were determined on day 4 by preparing Giemsa-stained thin blood smears and examining them under a microscope. Results are expressed as mean parasitemia ± SEM (*n* = 5). Significant differences in parasitemia levels compared to the UN are indicated as ^∗^*p* < 0.05, ^∗∗^*p* < 0.01, and ^∗∗∗^*p* < 0.001.

The suppressive antimalarial activity of *P. javanica* stem bark extract was evaluated using the standard 4-day suppressive test in PbANKA-infected mice (Figure 1). In the untreated control group, parasitemia reached 7.5 ± 0.8% by Day 4. ART, administered at a dose of 10 mg/kg, exhibited potent antimalarial efficacy, achieving a 90.7% inhibition of parasitemia, which was highly significant compared to the untreated group (*p* < 0.001). Treatment with *P. javanica* extract resulted in a dose-dependent reduction in parasitemia, with inhibition rates of 13.3%, 36.4%, and 52.5% observed at doses of 100, 200, and 400 mg/kg, respectively. Statistical analysis indicated that the 200 mg/kg dose significantly reduced parasitemia compared to the untreated group (*p* < 0.05), while the 400 mg/kg dose demonstrated a greater and highly significant effect (*p* < 0.01). These findings suggest that *P. javanica* stem bark extract possesses moderate suppressive antimalarial activity, with higher doses yielding statistically significant efficacy. The results support the potential of *P. javanica* as a promising source of antimalarial compounds, warranting further pharmacological and phytochemical investigation.

### 3.4. Curative Antimalarial Activity of *P. javanica* Stem Bark Extract Against PbANKA

**Figure 2:**
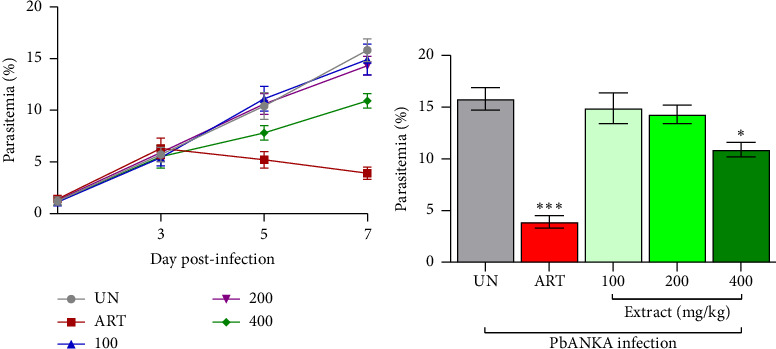
Curative antimalarial activity of *P. javanica* stem bark extract against PbANKA-infected mice. Mice were inoculated intraperitoneally with 1 × 10^7^ iRBCs of PbANKA on day 0 to establish infection. Oral treatments were initiated on day 3 post-infection and continued for four consecutive days (days 3–7). Treatment groups included *P. javanica* stem bark extract at doses of 100, 200, and 400 mg/kg, ART at a dose of 10 mg/kg, and an untreated group (UN) receiving vehicle (0.5% CMC). (a) Parasitemia was determined on days 3, 5, and 7 post-infections by preparing Giemsa-stained thin blood smears from tail vein samples and examining them under a light microscope. (b) Curative antimalarial activity on day 7 post-infection. Data are expressed as mean parasitemia ± SEM (*n* = 5). Significant differences in parasitemia levels compared to the UN are indicated as ^∗^*p* < 0.05 and ^∗∗∗^*p* < 0.001.

The curative antimalarial activity of *P. javanica* stem bark extract was evaluated in PbANKA-infected mice during established infections (Figure 2(a)). In the untreated control group, parasitemia increased progressively from 1.2 ± 0.4% on Day 1 post-infection to 15.8 ± 1.1% on Day 7, indicating uncontrolled parasite proliferation. ART, administered at 10 mg/kg, significantly suppressed parasitemia, achieving a 75.3% reduction compared to the untreated group (*p* < 0.001) on Day 7. Treatment with *P. javanica* stem bark extract at a dose of 400 mg/kg resulted in a 31.0% reduction in parasitemia, which was statistically significant relative to the untreated control (*p* < 0.05). However, the lower doses of 100 and 200 mg/kg did not produce significant reductions. The progression of parasitemia from Day 1 to Day 7 post-infection is shown in Figure 2(b), demonstrating the moderate curative effect of the extract and the superior efficacy of ART in controlling parasite growth. These findings indicate that *P. javanica* stem bark extract exhibits moderate curative antimalarial activity at higher doses and may serve as a complementary antimalarial agent for further development.

### 3.5. Assessment of Effective Doses of *P. javanica* Stem Bark Extract in PbANKA-Infected Mice

**Figure 3:**
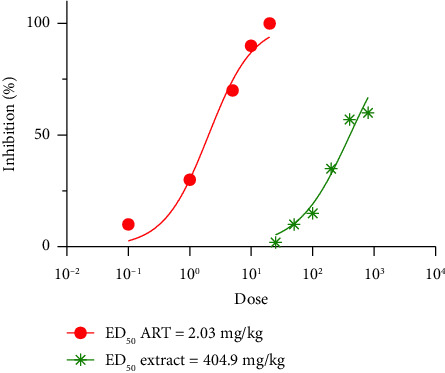
Effective doses of *P. javanica* stem bark extract and artesunate against PbANKA-infected mice. Mice were inoculated intraperitoneally with 1 × 10^7^ iRBCs of PbANKA on day 0 to establish infection. Oral treatments were administered once daily for four consecutive days (days 0–3). The treatment groups consisted of *P. javanica* stem bark extract at doses of 25, 50, 100, 200, 400, and 800 mg/kg, as well as ART at doses of 0.1, 1, 5, 10, and 20 mg/kg. Parasitemia levels were assessed on day 4 by preparing Giemsa-stained thin blood smears, and the percentage inhibition of parasitemia was calculated relative to the untreated control group.

The effective doses (ED_50_) of *P. javanica* stem bark extract and ART were determined through dose–response studies in PbANKA-infected mice (Figure 3). The ED_50_ values, defined as the doses required to achieve 50% inhibition of parasitemia, were calculated using nonlinear regression analysis of the respective dose-response curves. The ED_50_ of ART was 2.03 mg/kg, while the ED_50_ of *P. javanica* stem bark extract was 404.9 mg/kg, reflecting the greater potency of the standard drug relative to the plant extract. For the purpose of combination treatment studies, the ED_50_ values were rounded to 2 mg/kg for ART and 400 mg/kg for *P. javanica* extract to facilitate consistency in fixed-ratio dose calculations. These ED_50_ values provided a foundational reference for evaluating potential synergistic, additive, or antagonistic interactions between ART and *P. javanica* in subsequent combination therapy experiments. Overall, the findings highlight the moderate antimalarial efficacy of *P. javanica* stem bark extract as monotherapy and support its further exploration as an adjunctive agent in combination with conventional antimalarial drugs.

### 3.6. Antimalarial Activity of Combination Treatment

**Figure 4:**
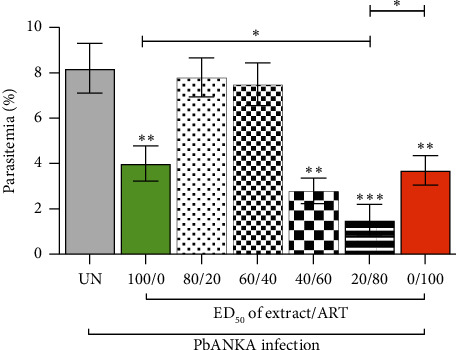
Antimalarial activity of combination treatment. The treatments were evaluated using fixed ratios based on the ED_50_ values of *P. javanica* stem bark extract (400 mg/kg) and ART (2 mg/kg). The combination treatments were prepared in fixed ratios of 100/0, 80/20, 60/40, 40/60, 20/80, and 0/100 (extract/ART). Oral treatments were administered once daily for four consecutive days (days 0–3) in PbANKA-infected mice. Parasitemia levels were determined on day 4 using Giemsa-stained thin blood smears, and the percentage inhibition of parasitemia was calculated relative to the untreated group (UN). Data are expressed as mean parasitemia ± SEM (*n* = 5). Significant differences in parasitemia levels compared to the UN are indicated as ^∗∗^*p* < 0.01 and ^∗∗∗^*p* < 0.001.

The antimalarial activity of combination treatments involving *P. javanica* stem bark extract and ART was evaluated at their respective ED_50_ values using fixed extract-to-drug ratios of 100/0, 80/20, 60/40, 40/60, 20/80, and 0/100 (Figure 4). In the untreated control group, parasitemia reached 8.2 ± 1.1% on Day 4 post-infection, indicating substantial parasite proliferation. Monotherapies using the 100/0 (extract only) and 0/100 (ART only) ratios resulted in 51.2% and 54.9% inhibition of parasitemia, respectively, both of which were statistically significant compared to the untreated group (*p* < 0.01). However, the 80/20 and 60/40 combination ratios did not produce significant inhibition compared to the untreated group, with calculated CI values of 12.8892 and 8.8907, respectively, indicating antagonistic interactions. In contrast, the 40/60 and 20/80 combinations exhibited 65.9% and 81.7% inhibition, respectively. These were statistically significant compared to the untreated group, with the 40/60 combination yielding *p* < 0.01 and the 20/80 combination yielding *p* < 0.001. The corresponding CI values were 0.8273 and 0.4412, indicating additive and synergistic interactions, respectively (Table 1). Notably, the 20/80 combination also demonstrated significantly greater antimalarial efficacy than either the extract or ART alone (*p* < 0.05). These findings suggest that combining *P. javanica* stem bark extract with ART, particularly at the 20/80 ratio, enhances antimalarial activity through synergistic effects. This supports the potential utility of integrating plant-derived compounds with standard antimalarial agents to optimize therapeutic outcomes, particularly in the context of emerging drug resistance.

### 3.7. MST of PbANKA-Infected Mice With Combination Treatment

The MST of PbANKA-infected mice was monitored over a 30-day observation period to assess the efficacy of *P. javanica* stem bark extract and ART as monotherapies and in combination (Table 2). In the untreated control group, the MST was 12.2 ± 1.9 days, indicating the fatal progression of malaria. Treatment with *P. javanica* extract alone (100/0) and ART alone (0/100) resulted in MSTs of 21.4 ± 2.4 days and 21.8 ± 2.4 days, respectively, both of which were significantly longer than the untreated group (*p* < 0.05). Combination treatments at fixed extract-to-ART ratios exhibited variable effects on survival. The 40/60 combination produced an MST of 21.6 ± 2.1 days, which was significantly extended compared to the untreated group (*p* < 0.05). Notably, the 20/80 combination achieved the longest MST of 28.6 ± 1.7 days, representing a highly significant improvement over the untreated group (*p* < 0.01) and a significant enhancement compared to either treatment alone (*p* < 0.05). These results demonstrate that combination therapy with *P. javanica* stem bark extract and ART, particularly at the 20/80 ratio, significantly prolongs survival in PbANKA-infected mice, suggesting a potential synergistic interaction. The findings underscore the therapeutic potential of integrating plant-derived extracts with conventional antimalarial agents to enhance treatment efficacy and address challenges related to drug resistance.

## 4. Discussion

This study evaluated the antimalarial activity of *P. javanica* stem bark extract as a monotherapy and in combination with ART against PbANKA in a rodent malaria model. The findings revealed dose-dependent efficacy of *P. javanica* stem bark extract in both suppressive and curative models, along with significant synergistic effects in combination treatments, highlighting the potential of *P. javanica* stem bark extract as a complementary antimalarial agent.

Hexane extraction of *P. javanica* stem bark is an efficient method for isolating nonpolar bioactive compounds, particularly lipophilic constituents such as quassinoids, alkaloids, and flavonoids. The moderate extraction yield (7.8% w/w) obtained in this study highlights the solvent's suitability for targeting these bioactive constituents, which are known to contribute to the pharmacological properties of *P. javanica* [12]. Moreover, hexane extraction may enhance the stability and bioavailability of these compounds, as nonpolar solvents can reduce oxidative degradation during the extraction process [11]. The selective nature of hexane also minimizes the coextraction of polar impurities, resulting in a concentrated crude extract enriched with bioactive constituents [18].

Acute toxicity testing indicated no adverse effects or mortality at doses up to 2000 mg/kg, indicating a wide safety margin for therapeutic applications. The absence of toxicity at the highest tested dose suggests that *P. javanica* stem bark extract is unlikely to pose acute health risks when used within this dose range [14]. The findings are consistent with the traditional uses of *P. javanica* in folk medicine, supporting its safety for further pharmacological applications [19]. The wide safety margin established in this study provides a strong foundation for future investigations into the therapeutic potential of *P. javanica* stem bark extract, including its use in combination therapies for malaria.

The evaluation of *P. javanica* stem bark extract in the 4-day suppressive and curative antimalarial models demonstrated promising dose-dependent activity. Notably, parasitemia inhibition exceeded 30% at higher doses, indicating potential antimalarial efficacy according to established benchmarks [20]. The threshold of 30% inhibition of parasitemia is widely recognized in preclinical studies as a benchmark for considering a compound or extract to have potential antimalarial activity [21]. This criterion is supported by several studies on plant-derived extracts, where inhibition rates above 30% in vivo rodent malaria models are indicative of biologically significant effects warranting further investigation [22, 23].

In the 4-day suppressive test, *P. javanica* stem bark extract exhibited significant dose-dependent inhibition of parasitemia, with 52.5% suppression observed at 400 mg/kg. Although less potent than ART, which achieved 90.7% suppression, the extract demonstrated moderate standalone efficacy. This result exceeds the 30% threshold, indicating the extract's potential to interfere with the early stages of parasite development in the blood. The observed suppressive activity is consistent with the presence of bioactive compounds, such as quassinoids, known to inhibit protein synthesis and disrupt mitochondrial function in *Plasmodium* parasites [11, 12]. Similarly, in the curative model, *P. javanica* stem bark extract at 400 mg/kg reduced parasitemia by 31.0%, while ART achieved 75.3% inhibition, meeting the threshold for potential efficacy. This activity suggests the ability of the extract to reduce parasitemia in established infections, complementing its suppressive effects. Lower doses of 100 and 200 mg/kg did not meet the 30% threshold, indicating that higher concentrations are necessary to achieve therapeutic levels of activity. The ability of *P. javanica* stem bark extract to surpass the 30% inhibition threshold in both suppressive and curative models underscore its potential as a source of antimalarial agents. Achieving this benchmark in both models is significant, as it demonstrates efficacy against both early-stage infections and established parasite loads. This dual activity is critical for developing therapeutic agents capable of addressing malaria at different stages of infection.

The ED_50_ values of *P. javanica* stem bark extract and ART were determined to be 404.9 mg/kg and 2.03 mg/kg, respectively. These values were used to establish fixed-ratio combination treatments. The combination of *P. javanica* stem bark extract and ART at various fixed ratios demonstrated significant enhancement in antimalarial activity, particularly at the 20/80 ratio (extract/ART). This combination achieved 81.7% parasitemia inhibition with a CI of 0.44121, indicating a synergistic interaction. Furthermore, mice treated with the 20/80 combination exhibited a significantly prolonged MST to 28.6 days, compared to monotherapies or untreated controls. In contrast, other ratios, such as 80/20 and 60/40, showed antagonistic interactions (CI > 1) or lacked significant improvements in efficacy. The unique synergism observed at the 20/80 ratio is likely due to complementary mechanisms of action between *P. javanica* stem bark extract and ART. *P. javanica* stem bark extract contains bioactive compounds, such as quassinoids, which disrupt mitochondrial function and inhibit protein synthesis in *Plasmodium* parasites [10, 11, 19, 24, 25], while ART generates reactive oxygen species (ROS) and targets calcium homeostasis [26]. At the 20/80 ratio, these mechanisms may work in concert, effectively targeting multiple pathways within the parasite.

The optimal dosing balance at the 20/80 ratio may also help minimize antagonistic interactions that could arise from higher concentrations of *P. javanica* stem bark extract, as seen in the 80/20 and 60/40 ratios. At higher doses, *P. javanica* stem bark extract could potentially compete with ART's mechanisms or saturate parasite uptake pathways, reducing the efficacy of the combination. Conversely, at lower ART concentrations, as in the 40/60 ratio, ART's potent ROS generation and calcium disruption mechanisms may not be fully exploited, leading to suboptimal antimalarial effects [23]. The 20/80 ratio, with a moderate dose of *P. javanica* stem bark extract (80 mg/kg) and a higher dose of ART (1.6 mg/kg), effectively balances these factors, reducing potential toxicity while enhancing therapeutic efficacy. This synergistic interaction at the 20/80 ratio highlights the potential of combining plant-derived extracts with existing antimalarial drugs to improve efficacy and address drug resistance. By lowering the required dose of ART, this approach may reduce the selective pressure for resistance development [26].

This study supports the use of plant-derived compounds, such as those found in *P. javanica*, as adjunctive agents in malaria therapy. Further studies are warranted to isolate and characterize the active phytochemicals, elucidate their mechanisms of action, and evaluate their efficacy in clinical settings. Additionally, long-term safety studies and pharmacokinetic analyses are necessary to establish the therapeutic potential of *P. javanica* stem bark extract in combination with standard antimalarial drugs.

## 5. Conclusion

This study demonstrates the antimalarial potential of *P. javanica* stem bark extract both as a standalone treatment and in combination with ART in PbANKA-infected mice. *P. javanica* stem bark extract exhibited dose-dependent suppressive and curative antimalarial activities, with significant parasitemia reduction at higher doses. The combination of extract and ART, particularly at a fixed ratio of 20/80 (extract/ART), demonstrated enhanced efficacy with synergistic interactions, achieving the highest parasitemia inhibition and prolonged survival compared to monotherapies. The findings highlight the potential of *P. javanica* as a source of complementary antimalarial agents that can enhance the efficacy of existing therapies. This approach could offer a promising strategy for overcoming drug resistance and improving malaria treatment outcomes. However, we acknowledge that these findings represent preliminary evidence. While CI analysis provides a useful quantitative indication of synergy, additional pharmacodynamic studies, such as isobologram construction, dose–effect surface modeling, and mechanistic evaluations, are required to conclusively establish a synergistic relationship. Future studies are also warranted to isolate and characterize the active compounds in *P. javanica* and further investigate their mechanisms of action and safety in clinical settings.

## Figures and Tables

**Table 1:** Combination index of AME combined with ART against PbANKA-infected ICR mice.

Antimalarial test	*P. javanica* stem bark extract/ART (fixed ratio)	CI value
ED_50_ combination	80/20	12.8892^b^
	60/40	8.89074^b^
	40/60	0.82731^a^
	20/80	0.44121^a^

^a^CI < 1, synergism.

^b^CI > 1, antagonism.

**Table 2:** MST of combination against PbANKA-infected mice.

Groups	*P. javanica* stem bark extract/ART (fixed ratio)	MST (day)
Untreated	0/0	12.2 ± 1.9

ED_50_ combination	100/0	21.4 ± 2.4^∗^
	80/20	11.6 ± 2.4
	60/40	12.0 ± 1.9
	40/60	21.6 ± 2.1^∗^
	20/80	28.6 ± 1.7^∗∗,a^
	0/100	21.8 ± 2.4^∗^

^a^ *p* < 0.05, compared to treatment with extract and ART alone.

^∗^ *p* < 0.05 and ^∗∗^*p* < 0.01, compared to untreated group.

## Data Availability

The data that support the findings of this study are openly available in Figshare at https://figshare.com/s/dfeb83d9f9eef87f7046, reference number 10.6084/m9.figshare.28032059.

## References

[1] Who (2023). World Malaria Report.

[2] Long B., MacDonald A., Liang S. Y. (2024). Malaria: A Focused Review for the Emergency Medicine Clinician. *The American Journal of Emergency Medicine*.

[3] Gozalo A. S., Robinson C. K., Holdridge J., Franco Mahecha O. L., Elkins W. R. (2024). Overview of *Plasmodium* Spp. and Animal Models in Malaria Research. *Comparative Medicine*.

[4] Alghamdi J. M., Al-Qahtani A. A., Alhamlan F. S., Al-Qahtani A. A. (2024). Recent Advances in the Treatment of Malaria. *Pharmaceutics*.

[5] Milong Melong C. S., Peloewetse E., Russo G., Tamgue O., Tchoumbougnang F., Paganotti G. M. (2024). An Overview of Artemisinin-Resistant Malaria and Associated Pfk13 Gene Mutations in Central Africa. *Parasitology Research*.

[6] Melong C. S. M., Peloewetse E., Russo G., Tamgue O., Tchoumbougnang F., Paganotti G. M. (2024). Correction to: An Overview of Artemisinin-Resistant Malaria and Associated Pfk13 Gene Mutations in Central Africa. *Parasitology Research*.

[7] Potterat O., Hamburger M. (2008). Drug Discovery and Development with Plant-Derived Compounds. *Progress In Drug Research*.

[8] Schmidt T. J., Khalid S. A., Romanha A. J. (2012). The Potential of Secondary Metabolites from Plants as Drugs or Leads against Protozoan Neglected Diseases-Part II. *Current Medicinal Chemistry*.

[9] Khan M. R., Kihara M., Omoloso A. D. (2001). Antibacterial Activity of *Picrasma javanica*. *Fitoterapia*.

[10] Prema W. C. P., Wong C. P., Kodama T. (2020). Three New Quassinoids Isolated from the Wood of *Picrasma javanica* and Their Anti-vpr Activities. *Journal of Natural Medicines*.

[11] Prema W. C. P., Wong C. P., Nugroho A. E. (2019). Two New Quassinoids and Other Constituents from *Picrasma javanica* Wood, and Their Biological Activities. *Journal of Natural Medicines*.

[12] Saiin C., Rattanajak R., Kamchonwongpaisan S. (2003). Isolation and *In Vitro* Antimalarial Activity of Hexane Extract from Thai *Picrasma javanica* B1 Stembark. *Southeast Asian Journal of Tropical Medicine and Public Health*.

[13] Janse C. J., Ramesar J., Waters A. P. (2006). High-efficiency Transfection and Drug Selection of Genetically Transformed Blood Stages of the Rodent Malaria Parasite *Plasmodium berghei*. *Nature Protocols*.

[14] Bedi O., Krishan P. (2020). Investigations on Acute Oral Toxicity Studies of Purpurin by Application of OECD Guideline 423 in Rodents. *Naunyn-Schmiedeberg’s Archives of Pharmacology*.

[15] Peters W., Portus J. H., Robinson B. L. (1975). The Chemotherapy of Rodent Malaria, XXII. The Value of Drug-Resistant Strains of *Plasmodium berghei* in Screening for Blood Schizontocidal Activity. *Annals of Tropical Medicine and Parasitology*.

[16] Peters W., Robinson B. L., Ellis D. S. (1987). The Chemotherapy of Rodent Malaria. XLII. Halofantrine and Halofantrine Resistance. *Annals of Tropical Medicine and Parasitology*.

[17] Nateghpour M., Farivar L., Souri E., Hajjaran H., Mohebali M., Motevalli Haghi A. (2012). The Effect of *Otostegia persica* in Combination with Chloroquine on Chloroquine-Sensitive and Chloroquine-Resistant Strains of *Plasmodium berghei* Using In-Vivo Fixed Ratios Method. *Iranian Journal of Pharmaceutical Research*.

[18] Ohmoto T., Koike K., Mitsunaga K., Fukuda H., Kagei K. (1989). Studies on the Constituents of Indonesian *Picrasma javanica*. III. Structures of New Quassinoids, Javanicins A, C and D. *Chemical & Pharmaceutical Bulletin*.

[19] Win N. N., Ito T., Ismail K. T. (2015). Quassinoids from *Picrasma javanica* Collected in Myanmar. *Journal of Natural Products*.

[20] Krettli A. U., Adebayo J. O., Krettli L. G. (2009). Testing of Natural Products and Synthetic Molecules Aiming at New Antimalarials. *Current Drug Targets*.

[21] Dechering K. J., Timmerman M., Rensen K. (2022). Replenishing the Malaria Drug Discovery Pipeline: Screening and Hit Evaluation of the MMV Hit Generation Library 1 (HGL1) against Asexual Blood Stage *Plasmodium falciparum*, Using a Nano Luciferase Reporter Read-Out. *SLAS Discovery*.

[22] Ounjaijean S., Somsak V. (2023). Synergistic Antimalarial Treatment of *Plasmodium berghei* Infection in Mice with Dihydroartemisinin and *Gymnema inodorum* Leaf Extract. *BMC Complement Medicine*.

[23] Ounjaijean S., Somsak V. (2020). Combination of Zingerone and Dihydroartemisinin Presented Synergistic Antimalarial Activity against *Plasmodium berghei* Infection in BALB/c Mice as *In Vivo* Model. *Parasitology International*.

[24] Chen J. J., Bai W., Lu Y. B., Feng Z. Y., Gao K., Yue J. M. (2021). Quassinoids with Inhibitory Activities against Plant Fungal Pathogens from *Picrasma javanica*. *Journal of Natural Products*.

[25] Win N. N., Ito T., Win Y. Y. (2016). Quassinoids: Viral Protein R Inhibitors from *Picrasma javanica* Bark Collected in Myanmar for HIV Infection. *Bioorganic & Medicinal Chemistry Letters*.

[26] Sinclair D., Zani B., Donegan S., Olliaro P., Garner P. (2009). Artemisinin-based Combination Therapy for Treating Uncomplicated Malaria. *Cochrane Database of Systematic Reviews*.

